# New Experiments and Observations on Hydrobromic Ether or the Bromide of Ethyl as an Anaesthetic
*Reprinted, with additions and corrections, from the *Journal of the American Medical Association*, November 21, 1885.


**Published:** 1886-04

**Authors:** Laurence Turnbull

**Affiliations:** Aural Surgeon to Jefferson Medical College Hospital; Physician to the Department of Diseases of the Eye and Ear, Howard Hospital, Philadelphia.


AMERICAN JOURNAL

OF

DENTAL SCIENCE.

VOL. XIX. THIRD SERIES.—APRIL, 1886. NO. 12.

ARTICLE I.

### NEW EXPERIMENTS AND OBSERVATIONS ON HYDROBROMIC ETHER OR THE BROMIDE OF ETHYL AS AN ANÆSTHETIC.*

BY LAURENCE TURNBULL, M. D., PH. G.

(Aural Surgeon to Jefferson Medical College Hospital; Physician to the Department of Diseases of the Eye and Ear, Howard Hospital, Philadelphia.)

The hydrobromic ether or bromide of ethyl was discovered by Serullas, in 1827, but received no special attention until Dr. Thomas Nunnelly, of Leeds, reported some experiments made with it on animals in 1849. Dr. Nunnelly brought the subject again before the profession, by a paper read at the meeting of the British Medical Association in 1865, in which, speaking of it in conjunction with another anæsthetic, he said he had for some time employed the one or the other in all the principal operations at the

*Reprinted, with additions and corrections, from the *Journal of the American Medical Association*, November 21, 1885.

Leeds General Eye and Ear Infirmary. This was at a time when chloroform held such complete sway in England, that no importance was attached to Nunnelly's experience or experiments. He had no one to follow him in using it; and we hear no more of it until 1876, when some experiments were made with it in France, by Rabuteau, on the lower animals; but evidently without a knowledge of the fact that this had been done previously in England by Nunnelly.

I then took the agent up without the knowledge of the experiments of Dr. Nunnelly. I had it made in Philadelphia by Professor Remington, and with two friends began experimenting in September, 1877; using it first on myself, and then upon my patients. When pure, bromide of ethyl is a volatile, colorless and almost uninflammable liquid, contrasting favorably in this respect with sulphuric ether, the highly inflammable and explosive properties of which are well known. It has a hot, but saccharine, taste; its specific gravity is 1.42, and it boils at 105.8° Fahrenheit. Its boiling point and density are therefore intermediate between those of chloroform and ether. After satisfying myself as to its efficiency and safety as an anæsthetic by experiments upon myself and others, I laid the subject before the Pennsylvania State Medical Society in 1878, and a record of ten cases, with my conclusions, was published in the volume of their transactions for that year. In August, 1879, I brought it before the British Medical Association at Cork; and in September of the same year, I presented a report of one hundred cases before the International Medical Congress at Amsterdam, to which I was a delegate from the American Medical Association. Up to March, 1879, when the second edition of my work on anæsthetics went to press, I had published a report of one hundred and twenty-five successful cases in quite a variety of surgical operations, and had not only employed it at my daily ear clinic, but also in the Jefferson Medical College Hospital; and I administered it in April, 1879, to a patient of Dr. Samuel W. Gross, at the public clinic, when he (Dr. Gross) removed a hyoid cyst from in front of the neck of a child. Dr. R. J. Levis, who was at this clinic, for the first time saw it employed, and became much interested in its use.

I thus compelled chemists to make it, by producing a demand for it; and gave them, through Dr. Green, a good formula for obtaining it free from impurities. I induced surgeons all over the country to try it, and especially the surgeons of this city, by bringing it in every way before their attention. The whole number of cases in which it has been employed by myself and friends was, up to June, 1880, some eight or nine hundred.

I cannot but feel disappointed that two deaths, not produced by it, should have been associated with it,* as advantage will be taken of such accidents by those having a prejudice against the ether, to condemn it on theoretical grounds.† In several instances recently the use of this anæsthetic has been attended with persistent vomiting, though in the thousands of cases in which it has been

*The bromide of ethyl as an anæsthetic, by Marion Sims, M. D., L L. D., *New York Medical Record*, April 3, 1880.

†In the discussion following the report of the fatal case by Dr. Sims to the New York Medical Society, Dr. Squibb undertook to account for the poisonous effects of bromide of ethyl by assuming it to be a loosely molecular article, easily decomposed; that thus its administration is prone to be followed by an impregnation of the system with bromine; and that if it remained as bromide of ethyl in the system it might not be harmful. This theory has been shown to be based on insufficient grounds. In the first place, Professor Jungk has shown that bromide is not "a loosely molecular article;" that in fact it is a very stable salt [for a salt it really is,] and very difficult of decomposition: much more difficult than chloroform. In the second place, the assumption that anæsthesia is due to a breaking up of the anæsthetic into its elements is nothing more than a hypothesis, and one, too, which has little or nothing to support it. The fact that one of the characteristics of bromide of ethyl is that it is perfectly unirritating to the bronchi, goes to show that it is not decomposed; if it were, the bromine in its composition, one of the most irritant of substances, would certainly manifest itself in its effects on the air passages.

employed, chiefly in Philadelphia, in not one single instance has it caused cerebral trouble, or any of the symptoms produced by the action of free bromine, which are as follows: when dogs are confined in an atmosphere of bromine vapor, they suffer a profuse secretion from the eyes, nostrils and fauces, with cough, hoarseness and dyspnœa. I have experimented upon frogs, cats, dogs, rabbits and various other animals, by subjecting them to an atmosphere highly charged with the vapor of hydrobromic ether, and in rare instances were there the effects as described above.

In the case of death under the employment of this agent in the hands of Dr. Levis, we do not think he was doing justice to it in subjecting the new anæsthetic to this most severe test. He knew the extreme debility of the patient, and that the most simple nervous shock would render him liable to death. Hundreds of patients have thus died. Again, when ordinary ether, chloroform, or other anæsthetics cause fainting; which was no doubt the result in this case, artificial respiration must be resorted to. We are reliably informed that in this instance the movement of the chest walls forced *the pus which was in this man's lungs into his bronchial tubes and suffocated him.* We are also very sorry that the valuable agent, nitrite of amyl, which has been found useful in such cases, was not employed.

The following report of the case will be of interest:

"PHILADELPHIA, June 2, 1880.

"Deputy Coroner Beam made an investigation of the circumstances, as reported in *The Times*, of the death of William Linderman, eighteen years old, of Schuylkill county, while upon the operating table at the Jefferson College Hospital under the influence of the new anæsthetic, bromide of ethyl, and about to be treated for stone in the bladder. He had been for about sixteen weeks under the care of Dr. R. J. Levis, one of the strongest advocates of the new anæsthetic, and was taken to the hospital by his direction. Linderman's health was very poor at the time. Dr. Ames, who administered the bromide, said an incision had not yet been made, but Dr. John B. Roberts said that there had. The patient was in such a condition that something had to be done, because he could not tide over the hot weather—96° to 98° in the shade.

"Dr. J. G. Lee, the coroner's physician, testified that he found the brain congested, *the lungs far advanced in consumption, and the kidneys and liver enlarged* and two large encysted stones in the bladder. His opinion was that they could not have been safely taken out. *Linder man could not have lived over a week or two at any rate.* Dr. Lee said further, that he had experimented with the the bromide on animals without bad results. In his opinion death resulted from exhaustion and prostration, the results of phthisis. The jury took the same view in their verdict."

As is well observed by Dr. Henry M. Lyman, "All experience shows that the administration of anæsthetics to certain patients is attended with danger. Even sulphuric ether may prove fatal if the kidneys are seriously damaged, and pulmonary disorganization is a well-known source of danger during the inhalation of anæsthetic vapor. The administration of chloroform to such a patient would be a very hazardous undertaking. The fatal results in these cases cannot be charged against the particular anæsthetic employed, but rather against the exhibition of any anæsthetic agent whatever."*

In some recent experiments on animals, I crowded four onces (the quantity stated to have been used by Dr. Sims,) upon a dog by means of a tin inhaler, until he became apparently dead, with no perceptible action of the heart or lungs; but the expression of his eye was clear, and the pupil was dilated; while there was no secretion from

*"Artificial Anæsthesia and Anæsthetics." Pages 220, 221.

the eyes or nostrils. The apparatus was removed in the space of four minutes, and he was exposed to the air, when at once he began to breathe, and by the end of six minutes, he had almost entirely recovered consciousness. The dog did not seem much inclined to move for ten or twelve minutes afterwards. While this dog was only partially under the influence of this anæsthetic, having at first caught the inhaling apparatus between his teeth, there was a good deal of rigidity, and slight tetanic movements of the extremities, but this was overcome by the free use of the ether.

Now, had we been using chloroform, just before we would have been ready to perform any experiments upon the animal, he would have been dead; and neither the removal of the anæsthetic nor exposure to air, would have been of any avail. Again, if Squibb's rectified and absolute ether had been employed, we must have super-saturated the animal, and been annoyed by the expectoration of large quantities of mucus, which in one recent experiment by me was followed by death. Then we frequently have seen tetanic convulsions, with great reduction of temperature, requiring several assistants to hold the patient, from the use of ordinary ether. The rapidity of the anæsthetic action of hydrobromic ether and its rapid elimination from the system by the lungs, are two of its chief merits for all operations that are not prolonged. *We recommend pure hydrobromic ether in operations not lasting over forty minutes.* For operations lasting one or two hours, we would advise the additional use of sulphuric ether; commencing after thirty or forty minutes' exhibition of the bromide of ethyl.

There is one great advantage in the use of this agent, that the administrator must attend to the anæsthetic all the time; he cannot watch the operation and forget the patient for a few seconds; his whole attention must be given to keep up its action. We believe that patients have sometimes been stifled by close pressure of the napkin, wet with the water present in ordinary ether, by the carelessness of the person giving it; whose attention has been given to the operation, rather than the patient.

As an anæsthetic in labor it has peculiar advantages, in that it is so rapid in its effects; the patient is comforted between the pains, but never passes into such a state of profound anæsthesia that she is not aroused by the expulsive effort, and has all her consciousness about her; and there are none of the depressing effects of ether or chloroform. It is also most valuable in these cases when it becomes necessary to change the position of the child; also in bringing forward the neck of the uterus into its proper position.* In none of my cases was there disturbance of the bowels, or pain in the back or head.

Miller, of Berne, speaks well of this obstetrical anæsthetic, which he has used in sixteen cases of primiparæ and six of multiparæ. Reddening of the face and acceleration of the pulse were frequently noted, thus giving the assurance that cerebral anæmia, as in the chloroform-syncope, need not be feared. The peculiar analgestic virtues were gratefully commented upon, especially by multiparæ. Hæckermann and Parnemann (Schmidt's Jahrbuecher No. 12, 1884,) express themselves in equally eulogistic terms of the anæsthetic in confinements.

To the country practitioner, who is obliged to extract teeth or perform any of the minor operations in surgery, it is a great boon, as it acts like nitrous oxide gas. It is well where a number of teeth are to be extracted, that a prop of hard wood attached to a string should be used; so as to prevent such an accident as once occurred in Philadelphia under the use of nitrous oxide gas: the swallowing of a prop of cork. It frequently happens in the use of hydrobromic ether, that when narcotism is not very profound, that the muscles of the patient become rigidly contracted. This condition occurred in a recent case, when we adminis-

*See pamphlet on the "Bromide of Ethyl as an Anæsthetic in Labor," by E. E. Montgomery, 12 pp. New York: W. Wood & Co., 1885.

tered ℥i of this anæsthetic; the operator's finger was caught and pinched, as also his forceps; and yet before operating we could touch the cornea with impunity. Although the impression passed away very rapidly, twelve teeth were extracted with entire success, the patient not feeling the pain, and promptly recovering consciousness.

In the following case the patient went under it very kindly. The patient was a man of very nervous temperament. With three drachms of hydrobromic ether, anæsthesia was produced without any struggling; and in four minutes from the time he had commenced to inhale it, the dentist had extracted ten teeth, and he had fully recovered consciousness, and without nausea; although he had just eaten a breakfast of solid food.

In a recent case of cataract extraction, the patient went beautifully under the influence of the anæsthetic, extraction was accomplished, and the patient recovered so as to be able to count fingers, yet owing to some strong coffee which she drank, from dyspeptic symptoms, or the swallowing of water soon after the operation, she became very sick at her stomach, and vomited for almost twenty-four hours; and yet the case did well. In a case of operation for torticollis, for a woman, she swallowed so much air with the ether, that as a consequence she complained of pain of a hysterical character, in lower part of the abdomen; the same which is often the result when nitrous oxide gas is inhaled, and too much air admitted.

In a letter received from the late Dr. J. Patterson Cassells, of Glasgow, a distinguished aurist and a surgeon to the celebrated Glasgow Infirmary, he writes that he has used a specimen of the hydrobromic ether, which I gave him at Cork, as vapor, in diseases of the middle ear, and has also employed it as an anæsthetic with success.

As I have before stated,* "*no anæsthetic can be used*

*See "A Presumable Ether-Death from Heart Failure," by John B. Roberts, M D., *Medical News*, September 27, 1884; by the same author, "Ether-Death," *Medical Times*, June 4, 1881. "Case of Death following the Inhalation of Chloroform," reported by P. L. Helsman, M. D., Albany [Ga.] *Medical News*, September 27, 1884.

*with absolute safety*:" all will kill. Chloroform kills, in round numbers, about one in every three thousand. Pure ether† is, next to nitrous oxide, the safest anæsthetic; only seventeen cases of death, and many of these doubtful, having been reported from its use. But it requires boldness and freedom in its administration; if slowly or ineffectually administered it is apt to produce a free secretion of bronchial mucus, which occasions troublesome coughing. If nitrous oxide is administered alone as a prelude to ether, the secretion of mucus is less troublesome, but there is a great amount of venous congestion and the tissues become gorged with blood, so that every incision tends to bleed. At times, also, wild excitement is produced by the gas. Some surgeons use the mixture which is known as A. C. E., which contains one part by measure of absolute alcohol, two of chloroform and three of Squibb's ether. This is not simply a mixture; the absolute alcohol,* 99.4 per cent., causes a solution of the other two, and they evaporate together. But the mixture should be administered freely from a cone of felt or flannel, with a paper covering, and the desired effect should be produced as rapidly as possible. The best results are by the agents which produce rapid effects, and which are as rapidly recovered from. No other has produced such rapid anæsthesia as the hydrobromic ether, and it is the most rapidly recovered from.

There are certain conditions of the system which forbid the use of anæsthetics. Again, there are certain of this class of agents that should not be employed in prolonged operations, as, for instance, the "bichloride of methyline," bichloride of ethedene, and bromide of ethyl. One or two deaths have followed the improper use of each of these agents, even when recommended by a committee appointed by the British Medical Association and by Sir

---

†Ether fortior, liquid, 94 per cent. of oxide ethyl, 6 per cent. of alcohol, and a little water.

*Specific gravity, .0716, at 77 deg., F.

Spencer Wells.† As the result of the observations and experiments with the bromide of ethyl, my conclusions have been that one hour is the longest time that a patient can remain under the influence of this anæsthetic with safety. In the case of potent remedies like morphine, atropine, hydrocyanic acid, etc., no one will attempt to ignore, or refuse to use such valuable remedies because in certain individuals and under certain conditions of the system, they produce death.

Can we in all cases rely on the experiments on animals as a true and absolute guide to determine our course in the human being? We think not; for it is a well-known fact that many animals eat plants which are deadly poisons to man, and certain anæsthetics are fatal to dogs.‡ Again, certain salts taken with impunity by man are poisonous to animals. The results of the prolonged experimental use of anæsthetics in the laboratory, even when of two hours'§ duration, cannot be taken as unquestioned as the results obtained by numerous careful observers on themselves and others. Clinical experience has now reached at least two thousand‖ well authenticated cases in which the bromide of ethyl has been employed with safety since 1880, when the two deaths were reported.

The following trials of this new anæsthetic were made to test its merits and to obtain personal experience of its

---

†Turnbull on "Artificial Anæsthesia," second edition, 1879.

Messrs. Regnauld and Villejean [*Lancet*, July 6, 1884,] have confirmed my statements [see page 65 of the last edition of my work,] that the so-called "chloride of methyline" is a mixture of chloroform and methylic alcohol.

‡Dr. B. A. Watson, Jersey City. "An Experimental Study of Anæsthetics." *Medical News*, p. 313, May, 1878. Method not given.

§"Two New Anæsthetics," by J. C. Reeve, M. D., Dayton, Ohio. *Cincinnati Lancet and Clinic.*

‖Dr. Chisholm, of Baltimore. *Maryland Med. Jour.*, January, 1883. Dr. Prince, of Jacksonville. *St. Louis Med. and Surg Jour.*, October, 1883, and Dr. L. Turnbull, of Philadelphia. *Medical Bulletin*, June, 1880.

effects. They were made by a gentleman very familiar with all the other anæsthetics, and his experience should be worthy of confidence. For the record of occurrences after loss of consciousness, and for care and attention during administration, he was indebted to his friends, Drs. Pilate and Conklin:

*First Experiment.*—March 14th. Four hours after eating a moderate breakfast he proceeded to inhale the bromide of ethyl, in the recumbent position; and from a bottle just opened, labeled "1 oz. bromide ethyl," about one-fourth of the contents was poured into an Allis ether inhaler. The first and immediate sensations upon inhaling it were a sharp pungent impression on the air passages, a sense of warmth rapidly extending, and exhilaration. With the second inspiration he felt a decided influence upon the brain, and began to talk; anxious to continue speaking as long as possible, and to state his sensations. A rapid beating in the ears is a constant symptom with him in taking chloroform, and immediately precedes entire loss of consciousness. He marked its presence now, and also its early appearance. It could not have been later than the third, or possibly the fourth, inspiration when he noted it, and this, as with chloroform, was the last sensation.

Upon opening his eyes after recovery from the anæsthetic, he immediately collected himself and could remember all; could talk clearly, and had no confusion of thought. He felt a slight sense of nausea and a feeling of languor. Eight minutes afterwards he got up and walked about without dizziness, and was confident he could have done so sooner. He did not attempt it sooner because he felt that sickness would ensue if he arose. The feeling of nausea remained until he commenced eating his next meal, about forty minutes later,

*Second Experiment.*—Pulse at beginning, 80; he having just ascended a flight of stairs. Two drachms administered. Symptoms began to be manifested after two respirations. Spoke of general warmth, pleasant sensations and beating in the ears. Anæsthesia produced in one minnte and a quarter; in another quarter minute it was profound, as tested by a knife point. Pulse during the first minute ran up to nearly 100, then fell during the next minute to about 70, feeble and intermittent. Pupils unchanged; normal; no struggling or excitement, but tetanic clutching of the inhaler so that it could be gotten away only with difficulty. The anæsthesia lasted one minute and a halt. He then awakened without mental confusion. Pulse seven to eight minutes later, 64. He was not satisfied with this experiment, particularly in regard to the irregularity and intermittence of the pulse: not a very assuring symptom in anæsthesia, and a result not agreeing with other observations. He had a suspicion from this fact, and from the nausea, that the specimen used was not pure. The bottle bore the name of a house which is always a guarantee of the good quality of medicines; but in the early period of manufacture of a new article, it would not be surprising if perfection was not immediately attained; he therefore obtained another specimen,* and one week after the above trial again inhaled it.

*Third Experiment.*—Being in the recumbent position, four hours after eating, one drachm, by measure, was poured into Allis' inhaler. He tried to take it slower this time, and count the respirations aloud to mark when conscious action ceased. He immediately felt the same grateful and pervading glow of warmth all over the body; counted to the seventh respiration; beating in the ears was again the last recognized impression. Pulse before, 80; at the end of the first minute, 120; one and a half minutes, at the rate of 100; at the end of two minutes, 78, no irregularity or intermittence; pupils unaffected; totally unconscious in one minute. Consciousness returned in three minutes.

It was his design to push the inhalation further this

---

* From the house of John Wyeth & Bro., Philadelphia.

time, and to test the muscular relaxation as well as to decide in regard to the irregularity of the pulse. Feeling that this had not been done, after about fifteen minutes he took it again.

*Fourth Experiment.*—Two measured drachms were poured on the inhaler, and he placed it over his mouth and nose. The impression was much stronger on the nose and air passages, and the first inspiration made him cough. He then counted to the third inspiration, and was gone. Pupils the same as before, unaffected; pulse before taking, 78; at the end of the first minute, 124; one and a half minutes, 100; and of two minutes, 78; no irregularity or intermittence. Anæsthesia in one minute. At the end of three minutes from the time of beginning he got up and walked across the room, and could have remained up. As an effort at prolonged anæsthesia this was not, therefore, a success. In eighteen minutes he was on his way driving to see a patient. He had not the slightest nausea after these two inhalations; felt, if anything, better than before.

*Fifth Experiment.*— His next trial of the agent and first attempt at administration was not satisfactory. The patient was a man aged about 50, a wiry, muscular fellow, of the type and build likely to give troublesome manifestions with any anæsthetic. He was placed on the table for an operation for hæmorrhoids, by Dr. Conklin, who had brought with him for the operation a large conical sponge, with which he was in the habit of giving the A. C. E. mixture. Upon this he poured two drachms of hydrobromic ether and placed it over the patient's mouth and nose. After one long, deep inspiration, his face became deeply flushed, and he soon began to talk and then to shout. More of the liquid was poured on the sponge; but his movements interfered with the inhalation of it with promptness; muscular rigidity then came on, and was marked; respiration was very nearly, if not quite, stopped for a time by tetanic spasm of the chest. These symptoms were almost as bad as are ever seen from ether, chloroform. or the mixed vapors. The Dr. had seen worse muscular action and rigidity, but this was as bad as generally met with. During this time the ether was rapidly added until the supply was exhausted (13 drachms,) and sufficient relaxation was not produced to make the operation feasible. No observations could be made, of course, of the patient's pulse. He recovered consciousness quite rapidly, as compared with other anæsthetics, and suffered no unpleasant after-effects.

This was not, of course, a fair trial of the remedy. The mode of administration was decidedly faulty. It is an ether, and must be given as an ether; and that this is imperative is the lesson to be learned by this failure.

My personal experience with hydrobromic ether fully sustains the observations of others as to its exceeding promptness of action, and the rapidity with which recovery from its effects takes place. It is also more pleasant to inhale than chloroform, which is not very unpleasant, and infinitely pleasanter than ether.

In my own experiments on animals I found that frogs placed in a watery solution of ethvlic bromide, become as completely anæsthetized as if they were immersed in an aqueous solution of chloroform.* Berger states to the Societe de Chirurgie (*Le Progres Medical,*) that he had been impressed by the rapidity with which these animals succumbed to its vapor. Terrillon administered the vapor of ethylic bromide to eighteen dogs without accident to any one of them.

Dr. Ott, of Easton, Pa., who has made thorough and scientific researches with the bromide of ethyl, experimenting upon frogs and rabbits, believes that the increased frequency of the pulse is due to stimulants of the accelerative nerves; or of the cardio-motor ganglia, and the dangers in administering the drug are less than those of nitrous oxide.

W. H. Hingston, Montreal, Canada, has used no other

---

*Op. cit. "Artificial Anæsthesia." Turnbull.

anæsthetic since commencing the use of bromide of ethyl. There is less resistance and struggling on the part of the patient. Vomiting is less frequent. It is eliminated from body more rapidly than any anæsthetic except laughing gas.

"Bromide of ethyl is one of the, and in some respects the, most valuable anæsthetic hitherto used."

In Terrillon's experiments, muscular relaxation occurred in human beings in two or three minutes; at times there was congestion of face, neck and upper part of the chest. The pupils did not contract, but were dilated. The pulse was always quickened, and every fresh dose caused fresh acceleration. Respiration was always hastened, and a hyper-secretion from the buccal and pharyngeal glands took place. Sensibility and consciousness returned with great rapidity; vomiting was not uncommon both during insensibility and sometimes for hours after. Verneuil, at the same meeting of the Societe de Chirurgie, stated that one patient, a woman, to whom he had given the vapor of ethylic bromide, was asleep in an instant; and Terrillon stated that anæsthesia may be produced in less than a minute. In our own experiments the shortest time necessary for primary anæsthesia was thirty seconds.

Dr. H. C. Wood found, by experiments upon animals, that if the vapor of ethylic bromide be given with moderation, anaesthesia may be produced without notable reduction of blood-pressure. He further observes (*The Th. Gazette*, June 15, 1885,): "After mixing with olive oil and agitating and distilling the liquid with this precaution we can obtain a safe and powerful anaesthetic, well adapted to cases of minor surgery which do not warrant the exhibition of ether and chloroform, and particularly eligible in obstetrical practice. In the experiments of Dr. C. C. F. Gay,* of Buffalo, the agent employed was evidently, from color and taste, impure, as was also that used by Dr. D. C. Wilkinson,† of Galveston, Texas. In Dr. I. C. Moore's

* ***Medical Record***, **July 17, 1880.**

† ***Medical Record***, **May 15, 1880.**

cases the ethyl was abandoned for ordinary ether, even when the insensibility had not passed off, owing to the exhibition of so-called bad symptoms, great excitement, with intense and persistent retching and vomiting, with venous engorgements. The article was stated to be pure, and was from Wyeth & Brother. The bromide of ethyl is costly, from the great care required in its preparation; and the great demand for it has caused many imitations to be placed on the market. The importance of its purity was at first so little understood that the original manufacturers did not take sufficient time to purify it, so that for a time the article contained carbon bromide ($C_2\ B_4$) and free bromine, phosphorus and bromoform. ‡ These were found in the specimen employed by Dr. Sims, which was a brown acrid liquid, with a pungent and disagreeable odor. Twenty drops of this given to a rabbit which had previously taken two grammes (thirty grains) of pure ethylic bromide without the slightest ill effect, produced irritation of the gastro-intestinal tract, followed by death in eighteen hours.*

That ethylic bromide may be employed with ease and success, has been abundantly proved by the experience of many observers. M. Bourneville has administered it to a large number of patients in the Salpetriere Hospital, for the arrest of paroxysmal hysteria and of epilepsy. He has also administered it daily by inhalation for fifteen or twenty minutes, with the fortunate result of considerably diminishing the frequency of the convulsive paroxysms. In several of these cases the temperature was depressed about half a degree centigrade during the act of inhalation. Immediately after the withdrawal of the anæsthetic the normal degree was recovered, and sometimes even surpassed. The pulse in about five hundred administrations was some-

---

‡ This can be prevented by mixing bromide of ethyl with five per cent. of olive oil, agitating and distilling the liquid successively.

* Dr. S. Wolff, *Am. Journal of Pharmacy*, May, 1880. The writer also obtained a portion of the same liquid from Dr. Wolff, and on comparing it with the specimen from Dr. Sims found it to be the same.

what accelerated during the period of inhalation. In six instances only was retardation observed. Respiration in like manner was almost always accelerated. A copious overflow of tears was nearly always remarked. The urine never contained either albumen or sugar, and the quantity of the liquid was not affected. Rigidity of the limbs and tremor involving the upper extremities, were sometimes noted. Daily inhalations for a period of two months exercised no unfavorable influence over the general process of nutrition; five patients found their weight increased during this period.

There are certain preparatory precautions which are necessary to the safe inhalation of the bromide of ethyl:

1. All tight-fitting garments in and about the neck and chest should be loosened.

2. The saturated ethyl vapor must be inhaled almost to the exclusion of atmospheric air. The best form of inhaler is a thick towel folded in the form of a cone, closed at the apex with a large pin; between the folds of the towel place a sheet of newspaper. The base of the cone must be wide enough to include both mouth and nose.

3. Instruct the patient, in advance, to make deep and long inspirations. In the cone place about one drachm, by measure, and at once cover the nose and mouth with it, and do not remove the cone until anæsthesia is produced, which will be in from twenty to thirty seconds.

The anaesthetic sleep will not last more than from two to three minutes. The patient retains the usual healthy color of lips and skin, and the pulse first becomes rapid, then slower and stronger as the narcosis becomes profound. The patient, as a rule, awakens suddenly and completely; but if there is nausea or much agitation, it is best for him to remain quiet and in a horizontal posture for some time.

Perhaps no operations are more painful than those on the eyes, eyelids, or eyeball, to a sensitive person, and there is no anaesthetic that I have found so applicable as bromide of ethyl in such operations. I recently administered it for the removal of a deep-seated tumor of the eyelid; the operation being performed by Dr. Hermann Knapp, of New York. The patient took the towel, with about two drachms of the ether, in her hands and applied it to her face, and in thirty seconds she was so completely anaesthetized that she was not conscious of one particle of pain until the tumor was entirely removed; she had no nausea whatever, or any other disagreeable symptom.

Again, in operations on diseased mastoid cells, I have employed it in some twenty cases with entire success, and in a very recent case, in which the whole bone was diseased and much of it had to be removed, the operation was of a most painful nature. I administered the bromide of ethyl to this patient, who was very much exhausted by profuse discharge from a large cancerous growth. The patient went under the influence of this anaesthetic with the most delightful effect, not suffering at all from the operation. and going to sleep after it without a bad symptom.

We have, in times past, heard a great deal of the injurious effects of bromides; and for a time, therefore, we gave the hydrobromic acid and ether with great caution, never exceeding thirty drops three times a day. But not so now; experience has taught us that we can use it, if well dilu‘ed, up to sixty drops three times a day without any injurious results. To obtain its full physiological effects in epilepsy, certain cases of pulsating tinnitus aurium, and in preventing the disagreeable cephalic symptoms occasioned by quinine and iron in these various nervous affections, we have found it at times very satisfactory. The salts of this agent, bromine, can be, and are, used with the greatest freedom in the form of bromide of potassium, sodium, and lithium, in doses of grs. xl—ʒ vi, given in six days without the least fear of its injurious effects upon the most delicate stomach; and relieving, as by a charm, convulsions, epilepsy, whooping cough, sleeplessness, headache, cerebral disturbance, tetanus, and all forms of mental derangement.

As is well observed by Dr. Chisholm:* "For office use I find the bromide of ethyl invaluable on account of its promptness, efficiency, the evanescent nature of the anæsthesia, the absence of nausea, and the perfect comfort with which patients operated upon can leave my office within a few minutes after the etherization."

Bromide of ethyl should never take the place of chloroform or sulphuric ether where any tedious operations are to be performed; but there is no reason why this useful anaesthetic should not be employed in all operations in minor surgery and in those on the eye, ear, throat and nose: having everything ready in advance, so that the patient shall be as short a time as possible exposed to the evil effect of an anaesthetic.

I conclude this brief paper by a statement of the conclusions arrived at in 1879. My favorable opinion remains unchanged at the present time, after using the article from 1878 to 1886 in all my office operations.

| | Minutes. | Seconds. |
|---|---|---|
| Shortest time taken to place a patient under the primary anæsthetic influence, . . . | 0 | 30 |
| Longest time, . . . . . . . . . . . . | 5 | 00 |
| Average time, . . . . . . . . . . . . | 1 | 30 |

I did not then advise that bromide of ethyl should be resorted to in protracted operations, and I never have employed it in any case longer than forty minutes, and have never used more than four ounces of the pure ether in one case.—*Dental Advertiser.*

1502 Walnut Street, Philadelphia, March 12, 1886.

---

* Maryland *Medical Journal*, January, 1883.

